# Vitamin D: a possible modifying factor linking obesity to vascular calcification in hemodialysis patients

**DOI:** 10.1186/s12986-017-0181-7

**Published:** 2017-03-17

**Authors:** Jwa-Kyung Kim, Mi Jin Park, Young Rim Song, Hyung Jik Kim, Sung Gyun Kim

**Affiliations:** 1 Department of Internal Medicine & Kidney Research Institute, Hallym University Sacred Heart Hospital, Pyungchon-dong, Dong-An Gu, 896, Anyang, Korea; 2 Department of Clinical Immunology, Hallym University Sacred Heart Hospital, Anyang, Korea

**Keywords:** Obesity, Vascular calcification, Percentage of body fat, Vitamin D deficiency, Hemodialysis

## Abstract

**Background:**

Obesity is a risk factor for increased cardiovascular disease. Whether vitamin D deficiency modifies this association is unclear. Here, we examined the association of obesity and vitamin D deficiency with vascular calcification score (VCS) in incident end-stage renal disease (ESRD) patients.

**Methods:**

A cross-sectional study was conducted with 213 ESRD patients. Vitamin D deficiency was defined as serum 25-hydroxyvitamin D (25(OH)D) levels below 10 ng/mL, and obesity was defined as a percentage of body fat (PBF) higher than the sex-specific median value in the cohort (>26.8% for men, >36.2% for women). VCS was measured by plain radiographic film of the lateral abdomen in the standing position.

**Results:**

Most ESRD patients (76.6%) had 25(OH)D deficiency at the start of dialysis. The prevalence of 25(OH)D deficiency was much higher in obese patients than non-obese patients, and it had significant inverse association with PBF (*r* = −0.315, *p* < 0.001). Abdominal aortic calcification was identified in 104 (48.9%) patients. VCS was significantly higher in obese population; 2.6 (0–23) for all patients, 4.2 (0–23) for obese and 1.0 (0–12) for non-obese patients (*p* < 0.001). Interestingly, vitamin D deficiency was associated with greater risk of a high VCS, especially in obese population [odds ratio (OR) 3.02, 95% confidence interval (CI) 1.09-9.38)], but not with non-obese patients (OR 1.82, 95% CI 0.56-5.60).

**Conclusion:**

The magnitude and direction of the association between obesity and the risk of vascular calcification may depend on an individual’s 25(OH)D level, a possible representative marker of cardiometabolic disturbance in ESRD patients.

**Electronic supplementary material:**

The online version of this article (doi:10.1186/s12986-017-0181-7) contains supplementary material, which is available to authorized users.

## Background

Obesity, often defined as an elevated body mass index (BMI), has been implicated in the development and progression of adverse cardiovascular outcomes in the general population. However, the long-term prognostic value of obesity may be reversed in specific populations, especially in patients with chronic illness such as coronary artery disease (CAD) and chronic kidney disease (CKD) [1]. Recent evidence has indicated that the protective effect of obesity may be conferred only to patients who have a healthy metabolic profile [2–4]. Namely, various metabolic risk factors such as insulin resistance, decreased muscle mass, lipid disorders, and chronic inflammation may influence the relationship between obesity and long-term outcomes in end-stage renal disease (ESRD) patients [5]. Indeed, obese ESRD patients who were metabolically healthy had a lower risk of mortality than normal weight ESRD patients who were metabolically healthy [4]. However, there was no mortality benefit in obese but metabolically unhealthy ESRD patients. In this context, there is an important limitation when using BMI to diagnose obesity. Patients with higher fat mass may have less favorable metabolic profiles than those with higher muscle mass, even if their BMI is same. Therefore, some authorities advocate a definition of obesity based on sex-specific values for percentage of body fat (PBF) that correspond to internationally recognized BMI cut-points for defining underweight, overweight and obesity [6].

Apart from the well-established role of vitamin D in CKD-mineral bone metabolism (MBD), more recent studies have established vitamin D as a representative parameter of cardiometabolic disturbance [7–9]. Low serum levels of 25-hydroxyvitamin D (25(OH)D) have been associated with higher prevalence of cardiovascular disease, diabetes, hypertension, obesity, and dyslipidemia [10–13]. Although the causal relationship between these associations has been unclear, their association may be evidentthe fact that obese population had low vitamin D levels is well-established. In fact, obesity is a risk factor for both vitamin D deficiency [14] and cardiovascular disease [15, 16]. A systematic meta-analysis showed a significant inverse association between BMI and vitamin D deficiency status in an adult population, and vitamin D supplementation improved cardiometabolic profiles [16].

Whether vitamin D levels can modify the link between obesity and cardiovascular risk is unclear. The purpose of this study was to evaluate the potential role of vitamin D for modifying the correlation between obesity, defined as elevated percentage of body fat (PBF), and vascular calcification score (VCS) in incident ESRD patients.

## Methods

### Study population and data collection

This study consisted of 269 ESRD patients who initiated dialysis between July 2011 and June 2015. We excluded patients who received oral vitamin D supplements in outpatient clinics during CKD management (*n* = 48), had a previous history of peritoneal dialysis or received kidney transplantation (*n* = 5), and who had an inserted pacemaker (*n* = 3), for a total of 213 patients included in the study. This study was approved by the institutional review board/ethics committee of Hallym University Sacred Heart Hospital, Anyang, Korea, and all study procedures are in adherence to the Declaration of Helsinki.

Data collected included age, sex, underlying cause of renal disease, comorbidities, systolic and diastolic blood pressure (BP), and various laboratory parameters. Biochemical analyses of hemoglobin, serum albumin, total cholesterol, high-density lipoprotein (HDL) cholesterol, low-density lipoprotein (LDL) cholesterol, triglycerides (TG), calcium, phosphate, intact parathyroid hormone (iPTH), 25(OH)D, and 1,25-dihydroxyvitamin D (1,25 (OH)2 D) levels were carried out at the start of dialysis. Levels of 25(OH)D were determined using an electrochemiluminescence method (Roche Cobas 8000 System, Tokyo, Japan). Levels ≥ 30 ng/mL were regarded as normal, while low levels between 10 – 30 ng/mL were considered to indicate vitamin D insufficiency, and very low levels < 10 ng/mL were categorized as vitamin D deficiency. In addition, more decreased 25(OH)D levels < 3 ng/mL were designated as severe vitamin D deficiency. Also, serum high-sensitivity C-reactive protein (hs-CRP) levels were measured using a Behring Nephelometer (BN) II Analyzer (Dade Behring, Newark, Del., USA) by a latex-enhanced immunoephelometric method. All other tests were performed according to the manufacturer’s instructions.

### Obesity evaluation and measurement of serum leptin

BMI was calculated by dividing the individual’s dry body weight (kg) by height squared (m^2^). Body composition data were obtained using a portable whole-body bioimpedance spectroscopy device (Body Composition Monitor; Fresenius Medical Care, Bad Homburg, Germany). The device provided objective data about fat mass, relative PBF, and fat tissue index (FTI) in which fat mass was normalized to the body surface area (m^2^). Obesity was arbitrarily defined as a PBF >26.8% for men and >36.2% for women in our cohort (sex-specific median value in our cohort). Also, serum leptin levels were measured using an enzyme-linked immunosorbent assay (ELISA) kit (R&D Systems, Wiesbaden, Germany) according to the manufacturer’s instructions.

### Vascular calcification score

The VCS was determined by plain radiographic film of the lateral abdomen in the standing position, as previously described by Verbeke et al. Briefly, abdominal aortic vascular calcification was graded on a 0 to 3 scale at each segment of the first through fourth lumbar vertebrae based on the severity of calcification [17]. A score of 0 denoted no aortic calcific deposits; 1 denoted small, scattered calcific deposits less than one-third of the longitudinal wall of the aorta; 2 denoted one-third or more, but less than two-thirds; and 3 denoted two-thirds or more. The anterior and posterior aortas were separately graded and the values were summed, resulting in a total score that ranged from 0 to 24. Particularly, VCS ≥ 7 was regarded as having severe VCS. All radiographic films were read by two experienced radiologists without knowledge of subjects’ clinical history.

### Statistical analysis

Statistical analysis was performed using the statistical package SPSS, version 24.0 (SPSS Inc., IL, USA). All variables were expressed as the mean ± SD or median with ranges, unless otherwise indicated. The Kolmogorov-Smirnov test was used to analyze the normality of distribution, and natural log values were used for skewed data including hs-CRP and serum leptin levels. Pearson’s correlation analysis was used to clarify the relationship between measured VCS, PBF, and various parameters including 25(OH)D. With multiple regression analysis, the influences of obesity and vitamin D deficiency on VCS were assessed. Multiple logistic regression analysis was performed to evaluate the determinants of significant vascular calcification development. A level of *p* < 0.05 was considered significant.

## Results

### Patient characteristics

This cross-sectional study included 213 consequent incident dialysis patients with a mean age of 63.7 ± 13.4 years; 31.9% (*n* = 68) were women and 64.7% (*n* = 138) had diabetes. Mean BMI and PBF were 23.8 ± 4.1 and 30.0 ± 10.5%, respectively. There was a definite disparity between BMI and PBF in incident ESRD patients; 26.8% patients were actually obese (diagnosed by PBF criteria) even though their BMI was less than 25 kg/m^2^, whereas 9.4% were non-obese (diagnosed by PBF criteria) even though their BMI was higher than 25 kg/m^2^ (Additional file 1: Figure S1).

**Fig. 1:**
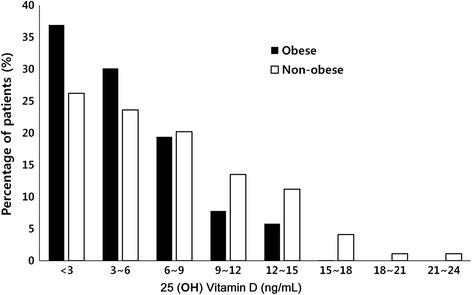
The distribution of 25(OH)D levels. The prevalence of 25(OH)D deficiency was much higher in obese patients than non-obese patients

**Fig. 2:**
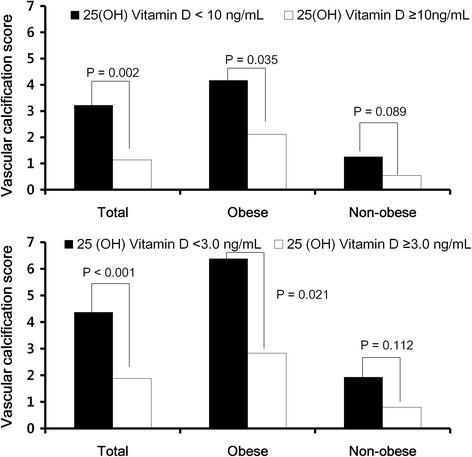
The differences of VCS according to the presence of obesity and 25(OH)D levels. Vitamin D deficiency was associated with greater risk of a high VCS especially with obese population, but not with non-obese patients (*upper*). The score gap became even larger when 25(OH)D levels were very low (*lower*)

Baseline characteristics of subjects with and without obesity are shown in Table 1. Compared to non-obese patients, obese patients were significantly older (*p* = 0.002), usually female in gender (*p* < 0.001), and diabetes was more common (*p* = 0.031). In addition, significant differences were observed in serum HDL (*p* = 0.035), LDL (*p* = .022), TG (*p* = 0.001), hs-CRP (*p* = 0.002), and leptin levels (*p* < 0.001). In addition, median serum 25(OH)D levels were significantly lower in the obese group. Figure 1 shows the distribution of 25(OH)D levels according to the presence of obesity. Most ESRD patients (*n* = 163, 76.6%) had 25(OH)D deficiency (<10 ng/mL) at the start of dialysis, and 34.2% (*n* = 73) had much lower 25(OH)D levels (<3 ng/mL, severe deficiency). Interestingly, the prevalence of 25(OH)D deficiency was much higher in obese patients than non-obese patients. No subjects had normal 25(OH)D levels (≥30 ng/mL) in the obese group (Fig. 2). In fact, serum 25(OH)D levels had a consistent, strong negative correlation with various representative parameters of adiposity such as PBF (*r* = −0.315, *p* < 0.001), serum leptin (*r* = −0.261, *p* = 0.002), and BMI (*r* = −0.209, *p* = 0.004) as well as the inflammatory parameter, hs-CRP (*r* = −0.256, *p* < 0.001). Serum 25(OH)D levels also correlated weakly with serum levels of iPTH (*r* = −0.16, *p* = 0.046). However, there was no correlation between 25(OH)D levels and serum calcium, phosphorus, or other metabolic parameters such as BP, glucose, or lipid profiles.

**Table 1:** Baseline characteristics

Clinical characteristics	Total (*n* = 213)	Obesity (+)^a^	Obesity (−)	
		(*n* = 109, 51.1%)	(*n* = 104, 48.9%)	p
Age (years)	63.7 ± 13.4	66.4 ± 13.0	60.8 ± 13.3	0.002
Gender, female, n (%)	68 (31.9)	47 (43.1)	21 (20.2)	<0.001
BMI (kg/m^2^)	23.8 ± 4.1	25.2 ± 4.3	22.2 ± 3.2	<0.001
BMI ≥ 25 kg/m^2^, n (%)	72 (33.8)	52 (47.7)	20 (19.2)	<0.001
Systolic BP (mmHg)	146.0 ± 20.8	147.3 ± 21.4	144.8 ± 20.3	0.416
Diastolic BP (mmHg)	82.1 ± 10.9	81.4 ± 11.1	83.4 ± 10.2	0.301
Coronary artery disease, n (%)	39 (18.3)	24 (22.0)	15 (14.4)	0.064
Causes of ESRD				0.044
Diabetes, n (%)	135 (63.8)	75 (68.8)	60 (57.7)	
Hypertension (%)	53 (83.6)	27 (24.8)	26 (25.0)	
Glomerulonephritis, n (%)	13 (6.1)	1 (0.9)	12 (11.5)	
Others, n (%)	12 (5.6)	6 (5.5)	6 (5.8)	
Body composition analysis				
PBF (%)	30.0 ± 10.5	37.5 ± 5.4	21.3 ± 7.8	<0.001
Fat tissue index (FTI)	9.7 ± 4.4	12.7 ± 3.5	6.5 ± 2.6	<0.001
Baseline laboratory findings				
Hemoglobin (g/dL)	8.9 ± 1.6	9.1 ± 1.5	8.7 ± 1.6	0.132
Serum albumin (g/dL)	3.57 ± 0.56	3.64 ± 0.54	3.49 ± 0.57	0.076
Total calcium (mg/dL)	8.1 ± 0.9	8.1 ± 0.9	8.0 ± 0.8	0.267
Corrected calcium (mg/dL)	7.9 ± 1.8	7.9 ± 1.8	7.8 ± 1.8	0.585
Phosphorus (mg/dL)	5.2 ± 0.83	5.2 ± 1.6	5.3 ± 2.0	0.400
Intact PTH	233.0 ± 171.5	236.9 ± 162.3	228.7 ± 182.0	0.743
Total cholesterol (mg/dL)	158.5 ± 42.9	162.8 ± 45.5	153.8 ± 39.7	0.152
HDL- cholesterol (mg/dL)	43.5 ± 14.4	41.3 ± 12.9	45.8 ± 15.5	0.035
LDL- cholesterol (mg/dL)	93.4 ± 33.7	99.1 ± 36.0	87.2 ± 29.9	0.022
Triglyceride (mg/dL)	124.6 ± 68.9	141.3 ± 73.2	106.6 ± 59.3	0.001
ln_hs-CRP	0.43 ± 1.24	0.68 ± 1.31	0.13 ± 1.10	0.002
25 (OH) vitamin D^a^	4.4 (2.2-8.0)	3.8 (2.2-6.7)	6.1 (2.6-10.1)	0.002
< 3 ng/mL	73 (34.3)	44 (40.4)	29 (27.9)	<0.001
3-10 ng/mL	90 (42.3)	53 (48.6)	37 (35.6)	
10-20 ng/mL	48 (22.5)	12 (11.0)	36 (34.6)	
≥ 20 ng/mL	2 (0.9)	0 (0.0)	2 (1.9)	
1, 25 (OH) vitamin D^a^	17.8 (12.1-22.7)	18.0 (12.1-25.5)	17.5 (12.8-21.6)	0.554
ln_leptin	1.86 ± 1.52	2.62 ± 1.07	0.86 ± 1.46	<0.001

^a^defined as a PBF(%) ≥ gender specific median value. *Abbreviations*: *BMI* body mass index, *WBC* white blood cell, *HDL* high-density lipoprotein, *LDL* low-density lipoprotein, *hs-CRP* high-sensitivity C-reactive protein^a^ median with IQR range

### Vascular calcification score and its relationship with serum 25(OH)D levels

Abdominal aortic calcification was identified in 104 (48.9%) patients and 33 (15.5%) patients had severe vascular calcification with VCS ≥ 7. The mean VCS was significantly higher in the obese population; 2.6 (0–23) for all, 4.2 (0–23) for obese, and 1.0 (0–12) for non-obese patients (*p* < 0.001). When obesity is defined by the BMI criteria, the VCS in the obese population was 3.4 (0–23), and 2.2 (0–15) for non-obese patients (*p* = 0.073). The prevalence of severe VCS was also higher in obese patients than non-obese (*p* < 0.001 by PBF criteria, *p* = 0.078 by BMI criteria). As shown in Table 2, VCS determination revealed a strong association with age (*r* = 0.370, *p* < 0.001), followed by serum leptin (*r* = 0.373, *p* < 0.001), hs-CRP (*r* = 0.339, *p* < 0 .001), PBF (*r* = 0.331, *p* < 0.001), 25 (OH)D (*r* = −0.207, *p* = 0.007), BMI (*r* = 0.163, *p* = 0.015), and serum phosphorus (*r* = −0.158, *p* = 0.034). There was no close relationship between VCS and iPTH or serum calcium levels in this study.

**Table 2:** Correlations between 25(OH)D, VCS and various metabolic risk profiles

	Age	SBP	DBP	BMI	PBF	Leptin	hsCRP	25(OH)D	1,25(OH)D	P	Alb	HDL	LDL	TG
VCS	0.370**	0.058	−0.118	0.163*	0.331**	0.373**	0.339**	−0.207*	0.078	−0.158*	0.083	−0.149	0.079	0.094
Age		0.124*	−0.288**	−0.155*	0.152*	0.152	0.166*	0.043	0.135	−0.261**	0.042	−0.096	0.068	−0.118
SBP			0.641**	0.018	−0.103	−0.079	−0.096	−0.005	0.041	0.046	−0.177*	0.189*	0.066	−0.023
DBP				0.024	−0.201*	−0.098	−0.163*	0.044	0.038	0.057	−0.072	0.186*	−0.072	−0.101
BMI					0.447**	0.517**	0.315**	−0.209*	0.014	−0.013	0.023	−0.332**	0.045	0.202*
PBF						0.671**	0.372**	−0.315**	−0.047	−0.055	0.158*	−0.182*	0.108	0.293**
Leptin							0.469**	−0.261*	−0.078	−0.043	0.237*	−0.365*	0.076	0.289**
hsCRP								−0.256**	0.086	−0.170*	0.112	−0.281**	−0.152	0.064
25(OH)D									0.275**	0.011	0.116	0.060	−0.011	−0.147
1,25(OH)D										−0.036	0.073	−0.096	0.042	0.123
P											0.003	−0.003	0.059	0.181
Alb												−0.097	−0.117	−0.008
HDL													0.078	−0.330*
LDL														0.264*

*p < 0.005, **p <0.001

Interestingly, in both the obese and non-obese groups, the VCS was significantly dependent on serum 25(OH)D levels. Figure 2 illustrates differences in VCS measured relative to the presence of 25(OH)D deficiency. In obese, non-obese and all patients, the VCS was higher in patients with 25(OH)D deficiency. Particularly, the difference was more clearly observed in the obese population than the non-obese group (Fig. 2, upper). The score gap became even larger with severe 25(OH)D deficiency (Fig. 2, lower). Similarly, observed (O) and expected (E) frequencies of presence or severe vascular calcification became significantly higher with increasing severity of low 25(OH)D levels. These findings were more marked in the obese population (Additional file 2: Table S1).

**Fig. 3:**
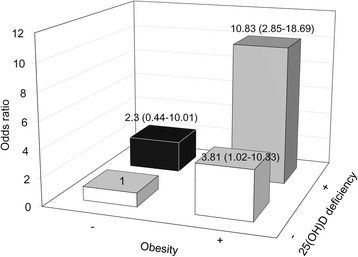
The ORs for the prevalence of severe VCS relative to the presence of obesity and 25(OH)D deficiency. When non-obese and non-25(OH)D-deficient patients serve as the reference group, obese patients with severe vitamin D deficiency had 10.83-fold increased risk for severe VCS (95% CI 2.85-18.69)

In a multivariate logistic regression model adjusted for age, sex, diabetes, systolic BP, previous history of CAD, and elevated HDL cholesterol, TG, calcium and phosphorus levels, patients with 25(OH)D deficiency had an increased tendency to develop vascular calcification as well as severe vascular calcification. However, the harmful effect of low vitamin D level was statistically significant only in the obese population. In the non-obese group, the modifying effect of 25(OH)D deficiency on vascular calcification was unclear (Table 3). The OR for the prevalence of severe VCS was the highest in cases of obesity and 25(OH)D deficiency, with non-obese and non-25(OH)D-deficient patients serving as the reference group (OR 10.83, 95% CI, 2.85 - 18.69) (Fig. 3).

**Table 3:** The effect of 25(OH) deficiency on the presence of VCS and severe vascular calcification: a multivariate logistic analysis^a^

	Vascular calcification			
	Presence (VCS ≥ 1)		Severe (VCS ≥7)	
	OR (95% CI)^a^	P	OR (95% CI)^a^	P
Total patients				
25(OH)D deficiency				
- Absence	1	-	1	-
- Presence	1.52 (0.62-3.73)	0.255	1.78 (0.77-4.55)	0.110
Obese patients				
25(OH)D deficiency				
- Absence	1	-	1	-
- Presence	3.02 (1.09-9.38)	0.034	5.75 (1.71-18.29)	0.005
Non-obese patients				
25(OH)D deficiency				
- Absence	1	-	1	-
- Presence	1.82 (0.56-5.60)	0.298	1.11 (0.07-10.95)	0.085

^a^ adjusted with age, sex, diabetes, systolic BP, previous history of CAD, and elevated HDL cholesterol, TG, calcium and phosphorus levels (for total patients, PBF was further adjusted)

## Discussion

In this cross-sectional study of 213 ESRD patients, we found that: 1) at the start of dialysis, most patients (76.6%) had 25(OH)D deficiency and more severely decreased 25(OH)D levels were also frequently observed; 2) obesity, defined as elevated PBF, was closely associated with a higher VCS and low serum 25(OH)D level; and 3) serum 25(OH)D affected the relationship between obesity and the risk of vascular calcification, such that vitamin D deficiency was associated with greater risk of a high VCS, especially with obese population, but not with non-obese patients. These findings suggest that the magnitude and direction of the correlation between obesity and the risk of vascular calcification may depend on an individual’s 25(OH)D level, a possible representative marker of cardiometabolic disturbance. In our knowledge, this is the first report to elucidate the potential role of 25(OH)D deficiency for aggravating the association between obesity and cardiovascular adverse outcome in incident ESRD patients.

With increasing numbers of obese dialysis patients, mortality for these patients was expected to be much higher than that of non-obese patient. However, contrary to the expectation, epidemiologic studies have shown the protective benefit of obesity in the ESRD population [1]. Although the exact reasons of this reverse epidemiology have been unclear as of yet, poor nutritional status or protein-energy wasting are strong predictors of worse outcomes in the CKD population [18]. Recent studies have found that the benefit of obesity could be conferred only to metabolically healthy obese population [2, 3]. In fact, with 4,374 CKD patients, Hanks et al. reported that overweight or obese patients who were metabolically healthy had lower risk for mortality as compared with metabolically healthy normal weight individuals [4]. However, there was no mortality benefit in metabolically unhealthy obese patients compared to metabolically healthy normal weight patients. It is very important to point out that an individual’s metabolic profile could be a potential and very decisive factor for determining adverse cardiovascular outcomes in patients with reduced kidney function.

In the general population, metabolic risks are often assessed in accordance with the criteria for metabolic syndrome. However, as more than half of ESRD patients have diabetes, which is one important criteria of metabolic syndrome, assessment of an individual’s metabolic risks while considering only traditional metabolic components such as BP, glucose, TG, and HDL levels has an important limitation in the ESRD population. Recently, vitamin D has been regarded as a possible representative marker of cardiometabolic disturbance [7]. Various observational studies have found a close relationship between vitamin D deficiency and numerous adverse extra-skeletal complications, including cardiovascular disease, metabolic syndrome, autoimmune disorders, neurodegenerative disorders, and cancer [8]. Moreover, vitamin D physiology may differ between subjects with obesity and non-obese individuals [19, 20]. A recent meta-analysis identified a significant inverse relationship between BMI and vitamin D status in an adult population [19]. Correction of vitamin D deficiency improved cardiometabolic profiles in adults with obesity [20]. However, there is limited data for evaluating the role of vitamin D as a metabolic risk factor in ESRD patients. Moreover, whether vitamin D status could affect the link between obesity and vascular calcification, especially in atherosclerosis-prone ESRD population remains unclear.

First of all, our data showed significantly much higher prevalence of 25(OH)D deficiency in our cohort compared to previous data. Wolf et al. reported that 18% of ESRD patients have 25(OH)D deficiency at the start of dialysis [21]. However, in our data, the prevalence was 76.6%, and the difference of the prevalence of much lower 25(OH)D levels (<3 ng/mL, severe deficiency) was more obvious. Although various hypotheses may be possible, but racial difference may be one of the important factors for the difference. In fact, Wolf also found significantly lower 25(OH)D levels in black subjects compared to white subjects [21]. Moreover, the prevalence of vitamin D deficiency or insufficiency has been reported to be significantly higher in ethnic minorities than the white population [19, 22].

But, consistent with previous data, 25(OH)D levels showed a strong relationship with various markers of obesity. Particularly, PBF had the highest correlation with 25(OH)D deficiency. Although BMI also had a negative association with 25(OH)D, the power was much less than PBF. Rather, adipokine levels, such as leptin, and inflammatory parameters, such as hs-CRP, showed a stronger relationship with 25 (OH)D levels than BMI. Interestingly, there was no significant relationship between 25(OH)D levels and other well-known metabolic components such as BP, HDL, TG, and glucose. Our data suggest that serum 25(OH)D could be a potential candidate marker for assessing metabolic risk, independent to the well-established metabolic syndrome components. Although low vitamin D levels in obesity has been reported in several previous data [15, 19, 23], its causes are not fully elucidated yet. The link between increased PBF and low vitamin D levels may involve chronic low grade inflammation associated with adipose tissue, as our data shows. Supporting this, recent data showed that total body vitamin D store are significantly greater in obese women than normal weight control, suggesting that the enlarged adipose mass in obese individuals serves as a reservoir for vitamin D and that the increased amount of vitamin D required to saturate this depot may predispose obese individuals to inadequate serum 25(OH)D [24].

As expected, PBF was closely associated with VCS. Jensky et al. reported that a 1 SD increment in abdominal or visceral fat was associated with a 1.6- and 1.5–fold increase, respectively, in risk for the presence of thoracic aortic calcium [25]. Similarly, with non-dialysis CKD patients, Cordeiro et al. showed that abdominal visceral fat is associated with coronary artery calcification scores [26]. However, the strength of our study is that we further investigated the detailed association according to the presence of 25(OH)D deficiency. Our data shows that higher PBF might confer significantly higher risk of increased VCS when 25(OH)D deficiency is also found. Particularly, for much lower 25(OH)D levels, higher and more severe vascular calcification was observed. However, interestingly, the modifying role of 25(OH)D levels was only relevant with the obese population. In the non-obese group, the effects of low vitamin D levels were not definite. The mechanisms explaining these observations are still unclear. But one possible explanation is that the potential harmful effect of low vitamin D may be more exaggerated in obese patients, as obese patients usually have higher systemic inflammation and advanced atherosclerosis. In fact, several observational data showed that vitamin D supplementation caused a significant reduction in body fat mass compared with placebo [27]. Similarly, High doses of vitamin D supplementation in diabetic patients was associated with significant decrease in arterial properties [28]. Maybe, the anti-inflammatory effect of vitamin D is associated with the changes of body fat mass and distribution. Also, obesity associated increased fibroblast growth factor 23 (FGF-23) and resultant vitamin D deficiency could potentiate the risk of vascular calcification. In fact, FGF23 has been shown to be associated with markers of insulin resistance, dyslipidemia and obesity, suggesting the FGF23 could be a cardiometabolic risk factor connecting obesity and higher cardiovascular complications [29]. Moreover, serum FGF23 levels are independently associated with higher levels of inflammatory markers in patients with CKD [30], which may play a major role in progression of vascular calcification. In fact, Mirza et al. reported that FGF 23 levels were higher in subjects with the increased adiposity compared with those without, and this potentially represent a novel pathway linking high FGF 23 to an increased cardiovascular risk [31]. Unfortunately, in this study, we could not measure other cardiometabolic risk factors except vitamin D levels.

There are some limitations in our study. First of all, we could not consider the seasonal variations of vitamin D levels. In general, 25(OH)D levels become lower in winter seasons due to insufficient sun exposure [32]. However, in this study, as most patients had vitamin D insufficiency or deficiency, the seasonal effect was thought to be minor. Also, a large number of patients (34.2%, *n* = 73) had very low 25(OH)D levels (<3 ng/mL, defined as severe vitamin D deficiency), and such low levels were not common in other disease populations. So the results may not extrapolate to other groups. Second, the causal relationship between low vitamin D and obesity is not clear in this study. Although most previous data suggest obesity causes vitamin D deficiency or insufficiency, the reverse relationship is also possible. However, as several previous studies showed that vitamin D supplementation could significantly reduce body fat mass and increase serum vitamin D concentration, in this study, we hypothesized that obesity could induce vitamin D deficiency or insufficiency. Future studies that evaluate the beneficial role of vitamin D supplementation in preventing development of vascular calcification or other cardiovascular events, especially in obese patients, may be needed. Third, we only had a single measurement of 25(OH)D, limiting our ability to establish the impact of vitamin D level changes during the study period. Last, even though we suggested the relationship between increased body fat mass and vascular calcification, we could not evaluate whole atherosclerotic profiles as well as early vascular complications such as endothelial dysfunction (actually, vascular calcification is a rather later step in atherosclerosis). Similarly, the well-established other biomarkers dealing with CKD-MBD, such as FGF23 or serum alkaline phosphatase levels were not measured in present study.

## Conclusions

25(OH)D deficiency is very common in incident ESRD patients. As the PBF became higher, VCS increases but 25(OH)D levels decreased. Compared to non-obese patients, obese patients had significantly higher VCS and severe vascular calcification. Particularly in the obese group, the association between PBF and VCS was dependent on 25(OH)D levels. Vitamin D deficiency was associated with greater risk of a high VCS, especially in obese population, but not with non-obese patients.

## Additional files

Additional file 1: VCS was higher in patients with 25(OH)D deficiency, particulary in obese patients. (TIF 781 kb)
Additional file 2: Table S1. Observed (O) and Expected (E) frequencies of the presence of vascular calcification according to obesity and various degrees of 25(OH)D deficiency. (DOC 37 kb)

## Abbreviations

25(OH)D: 25-hydroxyvitamin D
BMI: Body mass index
CAD: Coronary artery disease
CKD: Chronic kidney disease
CKD-MBD: Chronic kidney disease - mineral bone metabolism
ESRD: End-stage renal disease
hs-CRP: High-sensitivity C-reactive protein
PBF: Percentage of body fat
VCS: Vascular calcification score
