# Supplementary figures and images for: Vitamin D: a possible modifying factor linking obesity to vascular calcification in hemodialysis patients
Source: Nutr Metab (Lond). 2017 Mar 17;14:27. doi: 10.1186/s12986-017-0181-7 (PMC5356240; doi:10.1186/s12986-017-0181-7)

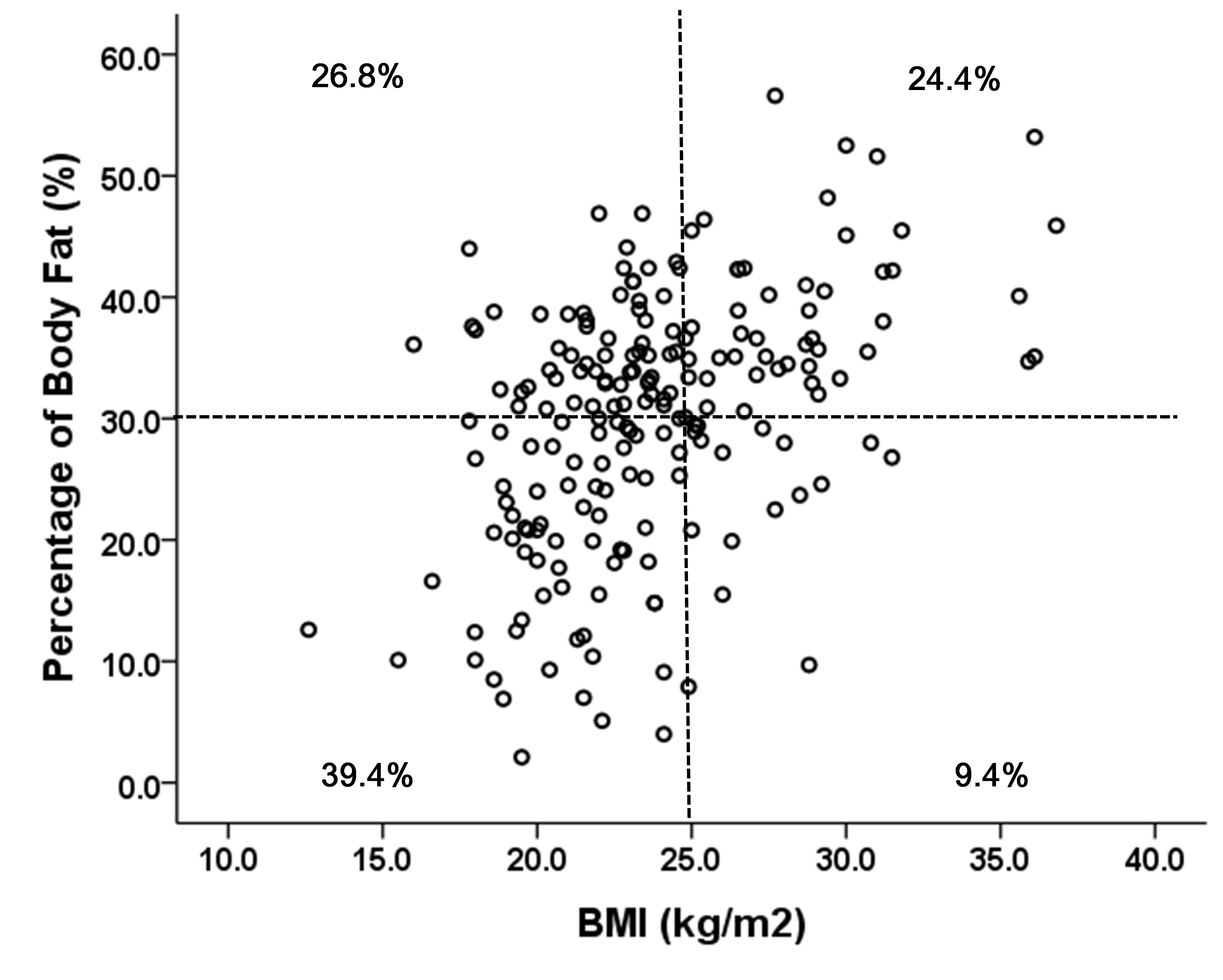

Supplement: Additional file 1: — VCS was higher in patients with 25(OH)D deficiency, particulary in obese patients. (TIF 781 kb) [file 12986_2017_181_MOESM1_ESM.tif]
