# Supplementary material for: Vitamin D: a possible modifying factor linking obesity to vascular calcification in hemodialysis patients
Source: Nutr Metab (Lond). 2017 Mar 17;14:27. doi: 10.1186/s12986-017-0181-7 (PMC5356240; doi:10.1186/s12986-017-0181-7)
Supplement: Additional file 2: Table S1. — Observed (O) and Expected (E) frequencies of the presence of vascular calcification according to obesity and various degrees of 25(OH)D deficiency. (DOC 37 kb) [file 12986_2017_181_MOESM2_ESM.doc]

Table 1. Observed (O) and Expected (E) frequencies of the presence of vascular calcification according to obesity and various degrees of 25 (OH)D deficiency

|  | | | Obese | | | |  | Non-obese | | | |
| --- | --- | --- | --- | --- | --- | --- | --- | --- | --- | --- | --- |
|  |  |  | 25(OH)D | | | |  | 25(OH)D | | | |
| VCS | Frequency | | <3 | 3-10 | 10-20 | ≥ 20 |  | <3 | 3-10 | 10-20 | ≥ 20 |
| None | O | | 10 | 19 | 6 | 0 |  | 17 | 27 | 29 | 1 |
|  | E | | 12.2 | 15.2 | 7.6 | 0.0 |  | 20.5 | 24.5 | 25.6 | 3.4 |
| ≥1 | O | | 35 | 32 | 7 | 0 |  | 12 | 10 | 7 | 1 |
| (presence) | E | | 35.1 | 32.2 | 6.7 | 0.0 |  | 9.9 | 9.5 | 9.0 | 1.6 |
|  | p = 0.068 | | | | | |  | p = 0.102 | | | |
| ≥7 | O | 18 | | 7 | 2 | 0 |  | 3 | 2 | 1 | 0 |
| (severe) | E | 12.5 | | 12.2 | 2.3 | 0.0 |  | 1.5 | 1.2 | 1.2 | 0.1 |
|  |  | p = 0.032 | | | | |  | p = 0.044 | | | |
